# Proton pump inhibitors increase the risk for hospital-acquired *Clostridium difficile* infection in critically ill patients

**DOI:** 10.1186/s13054-014-0714-7

**Published:** 2014-12-24

**Authors:** Jeffrey F Barletta, David A Sclar

**Affiliations:** Department of Pharmacy Practice, College of Pharmacy-Glendale, Midwestern University, 19555 N 59th Avenue, Glendale, AZ 85308 USA

## Abstract

**Introduction:**

Proton pump inhibitors (PPI) have been linked to *Clostridium difficile* infection (CDI) but there are few data specific to ICU patients. We evaluated duration of PPI exposure as a potential risk factor for hospital-acquired CDI in the ICU.

**Methods:**

This retrospective, case-control study was conducted using the Multiparameter Intelligent Monitoring in Intensive Care II database, a large publically available database of more than 35,000 ICU patients. Adult patients with CDI were identified using the ICD-9 code for *Clostridium difficile* listed as a secondary diagnosis. To be included, patients had to be present in an ICU for ≥48 hours prior to *Clostridium difficile* acquisition. These patients were then matched to patients without CDI using the ICD-9 primary diagnosis, age (+/−5 years) and SOFA score (+/−1). Successfully matched patients were reviewed for PPI exposure and other potential confounding variables for CDI. PPI exposure was characterized as short (<2 days) or long (≥2 days). Multivariate modeling was performed to identify independent risk factors for CDI.

**Results:**

There were 408 patients evaluated and 81% received a PPI. The percentage of patients who had a long exposure to PPIs was 83% in the CDI group compared to 73% with controls (*P* = 0.012). Upon inclusion of the following variables into a multivariate analysis (long PPI exposure, histamine-2-receptor antagonist administration, antibiotic administration, immunosuppression and study duration), long PPI exposure (odds ratio (OR) (95% confidence interval (CI) = 2.03 (1.23 to 3.36), *P* = 0.006) and antibiotic use (OR (95% CI) = 2.52 (1.23 to 5.18), *P* = 0.012) were identified as independent predictors of CDI.

**Conclusions:**

Proton pump inhibitors are independent risk factors for the development of CDI in ICU patients. This risk is particularly exposed after two or more days of therapy.

## Introduction

*Clostridium difficile* infection (CDI) is the leading cause of hospital-associated infectious diarrhea with considerable impact on length of stay and costs [1]. The prevalence of CDI in mechanically ventilated, intensive care unit (ICU) patients is 6.6% with most cases (69%) being diagnosed during the ICU admission [2]. The high frequency of CDI in critically ill patients is particularly concerning given the multiple risk factors that are present and the increased risk for adverse outcomes in this population.

Recently, proton pump inhibitors (PPIs) have been widely implicated as a significant risk factor for hospital-acquired CDI [3-9]. In one large database study of ICU patients, the odds ratio (OR) for CDI was significantly greater with PPI use compared to histamine-2-receptor antagonists (H2RA) (OR (95% confidence interval (CI) = 1.29 (1.04 to 1.64)). Infection-related risks with PPIs are believed to be greatest shortly after starting therapy [3,10-12]. One study evaluating the relationship between duration of PPI therapy and nosocomial CDI revealed a significant increase in risk after only two days of PPI use [3].

PPIs have become the most common modality for the provision of stress ulcer prophylaxis (SUP) in critically ill patients [13,14]. While PPI use for this indication is generally short-term, even an abbreviated exposure could lead to substantial increases in morbidity and overall hospital costs. The objective of this study was to further describe the relationship between PPI use and hospital-acquired CDI in critically ill patients and evaluate duration of inpatient PPI exposure as a risk factor for CDI.

## Methods

This case-control study was conducted using the Multiparameter Intelligent Monitoring in Intensive Care II (MIMIC II) database, version 2.6 [15,16]. This database is a large, publically available database that encompasses more than 35,000 patients admitted to the Beth Israel Deaconess Medical Center from 2001 to 2008. Beth Israel Deaconess Medical Center is a 620-bed tertiary academic medical center in Boston, MA, USA with 77 critical care beds [16]. The MIMIC II database provides a high-resolution record of time-stamped clinical variables, physiologic data, diagnoses and interventions that have been de-identified in a Health Insurance Portability and Accountability Act-compliant manner. The database was queried in August, 2013. Institutional Review Board approval was obtained (Midwestern University, AZ#754) prior to study initiation. The need for informed consent was waived.

Adult patients with CDI were first identified using the International Classification of Diseases, Ninth Revision (ICD-9) code for *Clostridium difficile* (008.45) listed as a secondary diagnosis. To be included, patients had to be present in an ICU for at least 48 hours prior to its acquisition. These patients were then matched to patients without CDI in a 1-to-1 ratio using the ICD-9 primary diagnosis, Sequential Organ Failure Assessment (SOFA) score (+/−1) and age (+/−5 years). Patients were excluded if *Clostridium difficile* was listed as a primary admitting diagnosis, if a successful match could not be obtained or if the medication record was missing or incomplete.

All successfully matched patients meeting inclusion/exclusion criteria were reviewed for demographics, medication history, comorbidities and other potential confounding variables for CDI. These included PPI exposure, H2RA use, antimicrobial therapy and immunosuppression. To characterize inpatient PPI exposure, two groups were formed based on the duration of PPI therapy, <2 days (short) or ≥2 days (long). These groups were formed based on previous research demonstrating an increase in risk for hospital-acquired CDI when duration approaches two days [3]. Classification and regression tree analysis was performed to confirm this cutoff. Antibiotic use was coded as yes if more than one dose of a systemic antibiotic was received. All drug exposures (PPI, H2RA, antibiotics) and durations of therapy were censored to the acquisition of CDI if applicable. Immunosuppression consisted of patients who received immunosuppressant drug therapy (for organ transplantation, lupus, HIV or arthritis), receipt of >10 mg prednisone equivalence or those with malignancy receiving chemotherapy. Study duration included the time from hospital admission to the acquisition of CDI (for CDI patients) or until hospital discharge (for control patients).

To determine the relationship between PPIs and CDI, patients were stratified into two groups based on the dichotomous presence of CDI. Confounding variables were compared between groups using univariate statistics. Student’s *t* test was used for continuous data that were normally distributed while Mann-Whitney *U* test was used for data that were skewed. Pearson’s chi-square or Fisher’s exact test were used as appropriate for dichotomous variables. Multivariate modeling was performed using conditional logistic regression with a backwards stepwise elimination procedure. Variables for inclusion into the model were selected using the results from the univariate analysis (that is, those with a *P* value <0.1) along with variables that were deemed to be clinically relevant to the acquisition of CDI. These variables were H2RA use, antibiotic use, immunosuppression and study duration. A *P* value <0.05 was used to determine statistical significance. IBM SPSS, version 19.0 (IBM Corp, Armonk, NY, USA) was used for all analyses.

## Results

**Figure 1:**
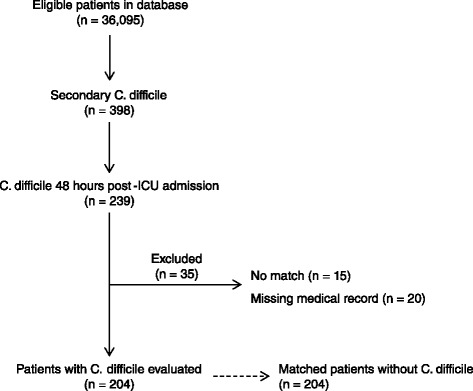
**Patient evaluation and stratification.**

There were 408 patients evaluated (Figure 1). The majority of patients were mechanically ventilated (73%) and admitted to either a cardiac ICU (51%) or a medical ICU (44%) (Table 1). For patients with CDI, the time from hospital admission to acquisition of *C.difficile* was 9.7 (2.4 to 56) days while the time from ICU admission to CDI was 8.3 (2 to 56) days. A total of 81% (332) patients received a PPI.

**Table 1:** **Demographics**

**Variable**	**All patients**
	**(n = 408)**
Age	69 ± 15
Gender (% male)	56% (229)
ICU type	
Cardiac/Cardiothoracic	51% (207)
Medical	44% (180)
Surgical	5% (21)
Classification of primary diagnosis	
Cardiovascular	24% (98)
Infection	23% (92)
Gastrointestinal	14% (56)
Respiratory	12% (50)
Neurologic	9% (36)
Cancer	6% (26)
Trauma	5% (20)
Renal	2% (10)
Venous thromboembolism	1% (6)
Miscellaneous	3% (14)
SOFA	6 (0 – 18)
Mechanical ventilation	73% (296)
Immunosuppression	30% (121)
Long PPI exposure (2 or more days)	78% (319)
PPI duration (days)	7 (0 – 96)
H2RA use	34% (138)
Long H2RA exposure (2 or more days)	28% (116)
H2RA duration (days)	0 (0 – 62)
Antibiotic use	90% (368)
Total number of antibiotics received	2 (0 – 8)
Study duration (days)	10 (2 – 99)

Data are presented as mean ± standard deviation, median (range) or % (n). ICU, intensive care unit; SOFA, Sequential Organ Failure Assessment; PPI, proton pump inhibitor; H2RA, histamine-2-receptor antagonist.

**Figure 2:**
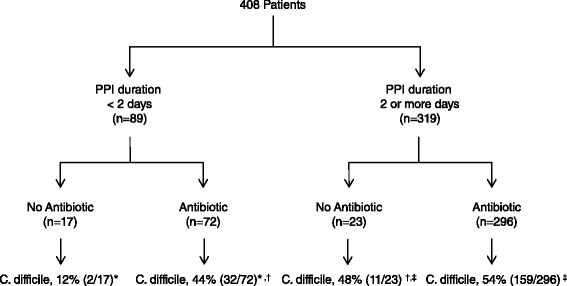
**The relationship between proton pump inhibitors, antibiotics and** ***Clostridium difficile.*** For pair-wise comparisons, ^*^ *P* = 0.013; ^†^ *P* = 0.777; ^‡^ *P* = 0.585.

PPI use was higher in patients who developed CDI compared to those who did not (Table 2). Specifically the percentage of patients who had a long exposure to PPIs (that is, two or more days) was 83% in the CDI group compared to 73% with controls (*P* = 0.012). Antibiotic use was also associated with CDI on univariate analysis. The relationship between CDI, PPIs and antibiotics is displayed in Figure 2.

**Table 2:** **Univariate analysis of confounding variables associated with** ***Clostridium difficile***

**Variable**	***C. difficile*** **- YES (n = 204)**	***C. difficile*** **- NO (n = 204)**	***P*** **value**
Age	68.6 ± 15	68.5 ± 15	0.949
Gender (% male)	55% (113)	57% (116)	0.765
ICU type			0.770
Cardiac/Cardiothoracic	51% (104)	51% (103)	
Medical	43% (88)	45% (92)	
Surgical	6% (12)	4% (9)	
Classification of primary diagnosis			1.00
Cardiovascular	24% (49)	24% (49)	
Infection	23% (46)	23% (46)	
Gastrointestinal	14% (28)	14% (28)	
Respiratory	12% (25)	12% (25)	
Neurologic	9% (18)	9% (18)	
Cancer	6% (13)	6% (13)	
Trauma	5% (10)	5% (10)	
Renal	2% (5)	2% (5)	
Venous thromboembolism	1% (3)	1% (3)	
Miscellaneous	3% (7)	3% (7)	
SOFA	6 (0 – 17)	6 (0 – 18)	0.798
Mechanical ventilation	74% (151)	71% (145)	0.506
Immunosuppression	28% (56)	32% (65)	0.329
Long PPI exposure (2 or more days)	83% (170)	73% (149)	0.012
PPI duration (days)	7 (0 – 56)	6 (0 – 96)	0.488
H2RA use	32% (65)	36% (73)	0.403
Long H2RA exposure (2 or more days)	26% (53)	31% (63)	0.272
H2RA duration (days)	0 (0 – 21)	0 (0 – 62)	0.474
Antibiotic use	94% (191)	87% (177)	0.020
Total number of antibiotics received	3 (0 – 7)	2 (0 – 8)	0.001
Study duration (days)	9.7 (2.4 – 55.7)	10 (2 – 99)	0.253

Data are presented as mean ± standard deviation, median (range) or % (n). ICU, intensive care unit; SOFA, Sequential Organ Failure Assessment; PPI, proton pump inhibitor; H2RA, histamine-2-receptor antagonist.

Upon inclusion of the following variables into a multivariate analysis (long PPI exposure, H2RA administration, antibiotic administration, immunosuppression and study duration), long PPI exposure (OR (95% CI) = 2.03 (1.23 to 3.36), *P* = 0.006) and antibiotic use (OR (95% CI) = 2.52 (1.23 to 5.18), *P* = 0.012) were identified as independent predictors of CDI (Table 3).

**Table 3:** **Multivariate analysis of confounding variables associated with** ***Clostridium difficile***

**Model**	**Covariates included**	**OR (95% CI)** ^*^	***P*** **value**
1	Long PPI exposure (2 or more days)	2.19 (1.27 – 3.78)	.005
	H2RA use	1.12 (0.70 – 1.79)	.628
	Antibiotic use	2.53 (1.23 – 5.23)	.012
	Immunosuppression	0.79 (0.51 – 1.23)	.297
2	Long PPI exposure (2 or more days)	2.08 (1.26 – 3.43)	.004
	Antibiotic use	2.53 (1.25 – 5.29)	.010
	Immunosuppression	0.79 (0.51 – 1.22)	.282
3	Long PPI exposure (2 or more days)	2.03 (1.23 – 3.36)	.006
	Antibiotic use	2.52 (1.23 – 5.18)	.012

^*^Odds ratios adjusted for study duration. OR, odds ratio; CI, confidence interval; PPI, proton pump inhibitor; H2RA, histamine-2-receptor antagonist.

## Discussion

*Clostridium difficile* is a highly prevalent nosocomial pathogen that represents a substantial burden to the health care system. Critically ill patients are at great risk for acquiring CDI given the many risk factors they are exposed to during their hospital admission. Historically, antibiotic use has been the primary drug-related culprit but acid suppressive therapy is increasingly being recognized as a probable cause. One study noted an increased risk for nosocomial CDI as the intensity of acid suppression increased [4]. Specifically, the ORs reported (compared to no acid suppression) were 1.53 for H2RA therapy, 1.74 for daily PPI therapy and 2.36 for more frequent administration. A second study evaluated the risk for CDI based on the duration of PPI therapy given most hospitalized patients receive daily PPI therapy for SUP [3]. In this study the OR (95% CI) for acquiring CDI was 1.14 (1.02 to 1.27) for each day of PPI therapy. Using classification and regression tree analysis, the risk for CDI increased when the duration of PPI therapy exceeded one day for patients with a prior hospital admission and two days in those without. However, only 26% of patients in this analysis were in an ICU, therefore, the validity of this model in critically ill patients is questionable. The current study was conducted to test the applicability of these thresholds in ICU patients.

Our results demonstrate that administration of a PPI for two or more days is associated with a two-fold increase in CDI. This is similar to data reported in non-ICU patients and confirms the previously identified thresholds for PPI duration and risk for CDI. Furthermore, we have revealed the risk for CDI caused by PPIs is similar to that observed with antimicrobial therapy. In fact, no difference in CDI incidence was noted in the cohort of patients who received antibiotics but had a short duration of PPI use (<2 days) compared to those who did not receive antibiotics but had a long duration of PPI use. A synergistic effect, however, was not observed when both antibiotics and long PPI therapy was provided. This differs from a previously published meta-analysis whereby the odds ratio for CDI was 1.97 for antibiotics alone and 1.82 for PPI alone but 3.44 when PPIs were administered with antibiotics [6].

The short duration of PPI exposure that is associated with CDI stimulates controversy regarding the true pathophysiologic mechanism of this relationship. One hypothesis is that increased gastric pH levels (caused by decreased acid secretion) facilitate the growth of pathogenic flora in the gastrointestinal (GI) tract. In addition, elevated gastric pH may also allow conversion from spores to vegetative cells that ultimately produce toxin [1]. The fact that maximal acid suppression with PPIs is not reached for several days after starting therapy yet the risk for infectious complications is greatest shortly after initiation bring reservation to this theory [12,17]. An alternative hypothesis relates to the immunomodulatory effects of PPIs and their ability to impair neutrophil activity [18-20]. In fact, in one study neutrophil bactericidal activity was decreased by 30% following a single dose of omeprazole [20]. Future research is required to elucidate the underlying mechanism.

There are few studies evaluating PPI therapy and CDI specific to the ICU. Beaulieu *et al*. reviewed medical records of medical ICU patients from the Project IMPACT database between March 2002 and May 2004 [21]. The incidence of CDI was 8.4 cases per 1,000 patient-days. Factors associated with CDI were receipt of clindamycin, macrolides, older age and female gender. PPI use was not identified as a significant risk factor. Shaughnessy, *et al.* conducted a retrospective cohort study in medical ICU patients to determine if hospital room assignment was associated with CDI [22]. In this analysis, prior room occupant with CDI was significant on multivariate analysis but neither PPI use nor antibiotic exposure was linked to CDI. Finally, MacLaren *et al*. conducted a large retrospective pharmacoepidemiologic study comparing GI hemorrhage and infectious complications in ICU patients who received either PPIs or H2RAs for SUP [9]. After adjusting for propensity score and covariates, the odds ratio for CDI was significantly higher with PPIs (OR (95% CI) = 1.29 (1.04 to 1.64)). Similar results were obtained in a propensity-matched analyses (PPI, 3.4% vs. H2RA, 2.6%, *P* = 0.002). This is the first analysis demonstrating increased risk for CDI with PPIs in ICU patients. Our results confirm these findings using an alternative large database of ICU admissions. Future, multicenter prospective trials are necessary to validate these conclusions.

PPIs have become the most common agents used for SUP. One large multicenter study of practice patterns across the United States and Canada revealed PPIs were chosen in 70% of patients who received SUP [13]. The short duration of PPI exposure that is linked to CDI could have tremendous clinical implications. Clinicians should investigate strategies to restrict PPI use for indications such as SUP. The reliance on local, institutional guidelines to curb practice relative to acid suppressive therapy appears to have minimal effect [13].

Several limitations are evident when interpreting the results of our study. First is the utilization of ICD-9 codes to identify outcomes and diagnoses. Limitations to ICD-9 coding have been previously reported (poor sensitivity, positive predictive value, and so on) [23]. However, a recent systematic review revealed their diagnostic predictability to be moderate to strong when used for CDI and they still remain the primary mechanism to extract outcomes from large database studies [24]. A second limitation is the case-control design and possibility for bias in the study groups. We attempted to account for this by matching patients using three criteria that included age, primary diagnosis and severity of illness. Nevertheless, some differences could have existed that were not detected. A third limitation is the possibility of confounding variables that were not examined in our multivariate analysis. Finally, outpatient utilization of PPIs could not be assessed.

## Conclusions

Duration of PPI use is significantly associated with the acquisition of CDI in critically ill patients. This risk is most evident when duration of therapy exceeds two or more days. ICUs should implement measures to restrict PPI use for indications such as SUP given the unlikelihood that therapy will be changed before the risk for infectious complications is apparent. Appropriately powered randomized controlled trials are necessary to confirm these findings.

## Key messages

- Duration of PPI use is significantly associated with the acquisition of nosocomial *Clostridium difficile*-associated diarrhea.
- This risk is evident after only two days of therapy.
- Clinicians should consider alternative forms of acid suppressive therapy (for example, histamine-2-receptor antagonists) for indications like stress ulcer prophylaxis.

## Abbreviations

CDI: *Clostridium difficile* infection
CI: confidence interval
GI: gastrointestinal
H2RA: histamine 2 receptor antagonist
ICD-9: International Classification of Diseases, Ninth Revision
ICU: intensive care unit
MIMIC II: Multiparameter Intelligent Monitoring in Intensive Care II
OR: odds ratio
PPI: proton pump inhibitor
SOFA: Sequential Organ Failure Assessment
SUP: stress ulcer prophylaxis
